# Laparoendoscopic single-site surgery compared with conventional laparoscopy for benign adnexal diseases: a systematic review and meta-analysis of randomized controlled trials

**DOI:** 10.3389/fmed.2026.1779247

**Published:** 2026-04-09

**Authors:** Peng Li, Lingli Hu, Debing Li, Yupei Lei, Weimin Xie

**Affiliations:** 1 Department of Anesthesiology, Affiliated Hengyang Hospital of Hunan Normal University & Hengyang Central Hospital, Hengyang, Hunan, China; 2 Department of Ultrasound, Affiliated Hengyang Hospital of Hunan Normal University & Hengyang Central Hospital, Hengyang, Hunan, China; 3 Medical Department, Affiliated Hengyang Hospital of Hunan Normal University & Hengyang Central Hospital, Hengyang, Hunan, China; 4 Department of Obstetrics, Affiliated Hengyang Hospital of Hunan Normal University & Hengyang Central Hospital, Hengyang, Hunan, China; 5 Department of Gynecology, Affiliated Hengyang Hospital of Hunan Normal University & Hengyang Central Hospital, Hengyang, Hunan, China

**Keywords:** benign adnexal diseases, conventional laparoscopy, laparoendoscopic single site surgery, meta-analysis, perioperative complications

## Abstract

**Background:**

Laparoscopic surgery has become the gold standard for the surgical management of benign gynecologic pathologies. Our objective was to assess the current evidence regarding the safety and efficacy of laparoendoscopic single-site surgery (LESS) in the treatment of benign adnexal diseases.

**Materials and methods:**

We comprehensively searched PubMed, the Cochrane Central Register of Controlled Trials (CENTRAL), Embase, and ClinicalTrials.gov from inception to 18 October 2025. We included randomized controlled trials (RCTs) comparing LESS with conventional laparoscopy (CL) for the treatment of benign adnexal diseases. Primary outcomes were the perioperative complication rate, postoperative pain, and cosmetic satisfaction. Secondary outcomes included operative time, estimated blood loss during surgery, hemoglobin drop, conversion to laparotomy, and length of hospital stay after surgery. All analyses were performed using random effects or fixed effects models. Clinical heterogeneity was explored using subgroup and sensitivity analyses.

**Results:**

We included eight articles reporting results from RCTs comparing LESS and CL in the final analysis. There were no significant differences between LESS and CL in terms of the perioperative complication rate (risk ratio (RR), 2.88; 95% confidence interval (CI), 0.70 to 11.78; *p* = 0.14) and postoperative pain scores at 6 h (weighted mean difference (WMD), −0.31; 95% CI, −0.75 to 0.13; *p* = 0.16), 24 h (WMD, −0.23; 95% CI, −0.46 to 0.00; *p* = 0.05), and 48 h (WMD, −0.24; 95% CI, −0.77 to 0.30; *p* = 0.39). There were also no differences in terms of operative time (WMD, 3.68; 95% CI, −0.81 to 8.17; *p* = 0.11), hospital stay after surgery (WMD, −0.13; 95% CI, −0.29 to 0.03; *p* = 0.11), estimated blood loss during surgery (WMD, −7.63; 95% CI, −31.83 to 16.57; *p* = 0.54), and hemoglobin drop (WMD, 0.18; 95% CI, −0.02 to 0.39; *p* = 0.08).

**Conclusion:**

This systematic review and meta-analysis provides evidence that LESS appears effective and safe for the treatment of benign adnexal diseases, as it is generally equivalent to CL in terms of perioperative outcomes.

**Systematic review registration:**

https://www.crd.york.ac.uk/PROSPERO/view/CRD42024608657, Identifier: CRD42024608657.

## Introduction

Laparoscopic surgery has become the gold standard for the surgical management of benign gynecologic pathologies. Reduced morbidity, improved aesthetics, shorter length of hospital stay, less pain, and more rapid recovery have been associated with minimally invasive surgical approaches compared to laparotomy (1, 2). Recent improvements in laparoscopic surgical techniques have led to the introduction of laparoendoscopic single-site surgery (LESS) to further reduce incisional morbidity, including complications such as vascular injury, postoperative hernia, and infection. With this technique, only one trocar is inserted into the umbilical region, and several instruments are introduced through this trocar. This offers a less invasive method compared to conventional laparoscopy (CL) for completing laparoscopic surgical procedures.

LESS has been reported to be feasible and comparable to CL across various surgical fields (3–5). More recently, several studies have evaluated the feasibility of LESS for adnexal surgery in benign diseases (6–9). Although an increasing number of studies have compared the surgical outcomes of LESS and CL for benign adnexal diseases, few are randomized controlled trials (RCTs), and the results are conflicting. Therefore, it remains unclear which surgery is more advantageous and should be recommended. This meta-analysis aimed to systematically search and analyze the available RCTs to compare the safety and efficacy of LESS with CL for benign adnexal diseases. This research was conducted and reported in strict adherence to the Transparency in the Reporting of AI Use (TITAN) criteria, with confirmation that no AI was employed during any stage of the research (11).

## Methods

### Search strategy and study selection

This meta-analysis was reported in accordance with the PRISMA (Preferred Reporting Items for Systematic Reviews and Meta-Analyses) (15) and AMSTAR (Assessing the Methodological Quality of Systematic Reviews) guidelines (16). The study protocol was registered on PROSPERO on 14 November 2024. To identify studies investigating the safety and efficacy of LESS and CL for benign adnexal diseases—a pre-specified planned analysis—we conducted a literature search of PubMed, the Cochrane Central Register of Controlled Trials (CENTRAL), Embase, and ClinicalTrials.gov from inception to 18 October 2025. The search strategy combined the following terms: “single incision OR single site OR single port OR single access OR single trocar” AND “laparoscop* OR laparoendoscop* OR coelioscop* OR celioscop* OR peritoneoscop* OR abdominoscop*” AND “ovarian cystectomy OR ovariectomy OR adnexectomy OR adnexal surgery OR salpingo-oophorectomy OR adnexal preservation OR adnexal disease OR adnexal mass OR adnexal tumor.”

We then included relevant studies that met the following criteria: (a) population: women with benign adnexal diseases, (b) intervention: LESS, (c) comparator: CL, (d) outcomes: perioperative outcomes, and (e) study design: randomized controlled trials (RCTs). We excluded the following studies: (a) non-original research articles, such as review articles, case reports or series, abstracts, letters to the editor, editorials, expert opinions, comments, and book chapters; (b) non-randomized observational studies; (c) studies lacking a comparison group; and (d) studies with no extractable or calculable outcome data. For studies published more than once, only the largest and most informative reports were included.

### Data extraction

When comparing the two surgical approaches, surgical safety was prioritized. Primary outcomes were the perioperative complication rate, postoperative pain, and cosmetic satisfaction. Secondary outcomes included operative time, estimated blood loss during surgery, hemoglobin drop, conversion to laparotomy, and hospital stay after surgery. Perioperative complications encompassed all adverse events occurring during the perioperative period, including intraoperative and postoperative complications. Postoperative pain was assessed using a Visual Analog Scale (VAS) or a Simple Numerical Scale (SNS). The scores ranged from 0 to 10, with “0” indicating “no pain” and “10” indicating “severe pain.”

In total, two authors independently extracted data from all eligible RCTs, and any discrepancies were resolved through consensus with a third author. The following data were extracted from each study: The first author’s name, year of publication, data source, number of patients in the LESS and CL groups, age, body mass index (BMI), follow-up period, and the outcomes of interest that were previously mentioned. For continuous outcomes, we extracted the sample size, mean, and standard deviation (SD). When studies reported the median (with range) instead of the mean (with SD), the mean and SD were estimated using validated methods (17). Studies providing no available data on the SD or range were excluded from this meta-analysis. For dichotomous outcomes, we extracted the total sample size per group and the number of events.

### Quality assessment

Furthermore, two independent authors evaluated the methodological quality of the RCTs included in this meta-analysis using the tool recommended by the Cochrane Collaboration (18). The risk of bias tool covers five domains of bias: randomization, intervention deviations, missing data, outcome measurement, and result reporting. Any disagreements during the quality assessment were resolved through discussion with a third author until consensus was reached.

### Statistical analysis

Statistical analyses were performed using Review Manager version 5.3.5 (Cochrane Collaboration, Oxford, United Kingdom), with a two-sided *p*-value of <0.05 indicating statistical significance. Continuous outcomes were analyzed using the weighted mean difference (WMD), and dichotomous outcomes were analyzed using the risk ratio (RR). A 95% confidence interval (CI) was used for all results. Heterogeneity among the included studies was measured using the chi-squared test and the *I*^2^ test. If the *p*-value was ≥0.10 or *I*^2^ ≤ 50%, indicating low heterogeneity, a fixed effects model was utilized for analysis; if the *p*-value was <0.10 or *I*^2^ > 50%, indicating significant heterogeneity, a random effects model was employed for analysis after clinical heterogeneity was excluded (19). Clinical heterogeneity was explored using subgroup and sensitivity analyses. As the number of studies included in this meta-analysis was fewer than 10, we did not use funnel plots to evaluate publication bias, in accordance with the Cochrane Handbook for Systematic Reviews.

## Results

### Search results

**Figure 1:**
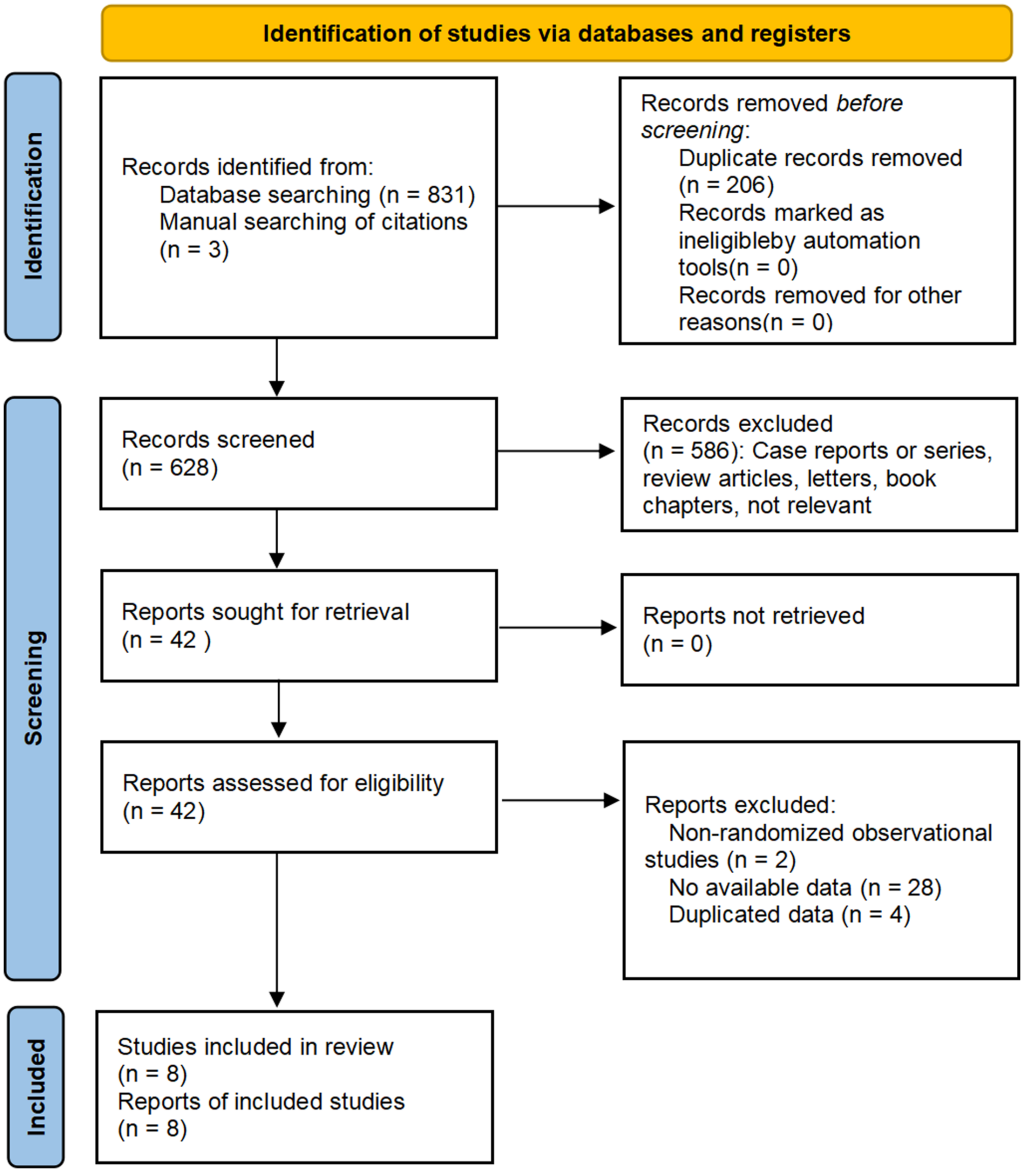
PRISMA flow diagram of the study selection process.

Following the established search strategy, a total of 834 records were retrieved, including 257 articles from PubMed, 103 from CENTRAL, 462 from Embase, nine from ClinicalTrials.gov, and three additional articles identified using reference lists. After removing duplicates and screening titles and abstracts based on the established criteria, 42 potentially relevant records were evaluated for eligibility. Of these, 34 records were excluded for the following reasons: Non-randomized observational studies (*n* = 2), no available data on outcomes of interest (*n* = 28), and duplicate data from overlapping publications (*n* = 4). The remaining eight articles reported results from RCTs comparing LESS and CL for benign adnexal diseases, met the inclusion criteria, and were included in the present meta-analysis (20–27). The PRISMA flow diagram of the study selection process is depicted in Figure 1.

All included studies were published in English and involved 525 patients between 2011 and 2024, with sample sizes ranging from 40 to 100. In all included studies, the same surgical team with extensive experience in both LESS and CL procedures performed all operations. The characteristics of the included studies are shown in Table 1. Baseline characteristics, including age and BMI, were comparable between the two groups in all included studies. A total of four studies were from South Korea (20, 25–27), one study was from Italy (21), one study was from the United Kingdom (22), one study was from Turkey (23), and one study was from France (24).

**Table 1:** Characteristics of the included studies.

Author, year	Location	Study center type	Sample size		Age (year)		BMI (kg/m^2^)		Duration of follow-up
			LESS	CL	LESS	CL	LESS	CL	
Schmitt et al., 2024 (20)	South Korea	Multicenter	33	30	29.5 ± 6.2	31.1 ± 7.2	21.4 ± 3.2	22.5 ± 3.3	4 weeks
Shin et al., 2019 (21)	Italy	Single-center	30	30	49.0 (20–73)	42.0 (15–73)	22.8 (17.6–37.0)	22.1 (18.2–30.0)	1 month
Yoo and Shim, 2013 (22)	United Kingdom	Single-center	20	20	55.1 ± 16.2	58.7 ± 10.8	25.1 ± 5.5	25.4 ± 4.8	2 months
Yoon et al., 2014 (23)	Turkey	Single-center	32	39	31.1 ± 8.35	29.9 ± 7.96	24.8 ± 3.69	23.4 ± 2.83	Not reported
Pelosi and Pelosi, 1991 (24)	France	Single-center	49	51	46 (18–84)	44 (21–69)	24.5 (17.6–38.3)	25.1 (17.3–42.4)	1 month
Yoon et al., 2010 (25)	South Korea	Single-center	31	30	36.5 ± 14.5	39.9 ± 15.8	21.1 ± 3.0	22.3 ± 3.0	4 months
Jung et al., 2010 (26)	South Korea	Single-center	38	35	33 (19–67)	39 (20–63)	23 (18–28)	24 (18–31)	1 year
Fader et al., 2009 (27)	South Korea	Single-center	28	29	30.3 ± 5.0	28.8 ± 5.2	20.4 ± 2.4	20.4 ± 2.4	3 months

LESS, laparoendoscopic single-site surgery; CL, conventional laparoscopy; BMI, body mass index. Data are expressed as numbers, means ± standard deviations, or medians (range).

### Risk of bias

**Figure 2:**
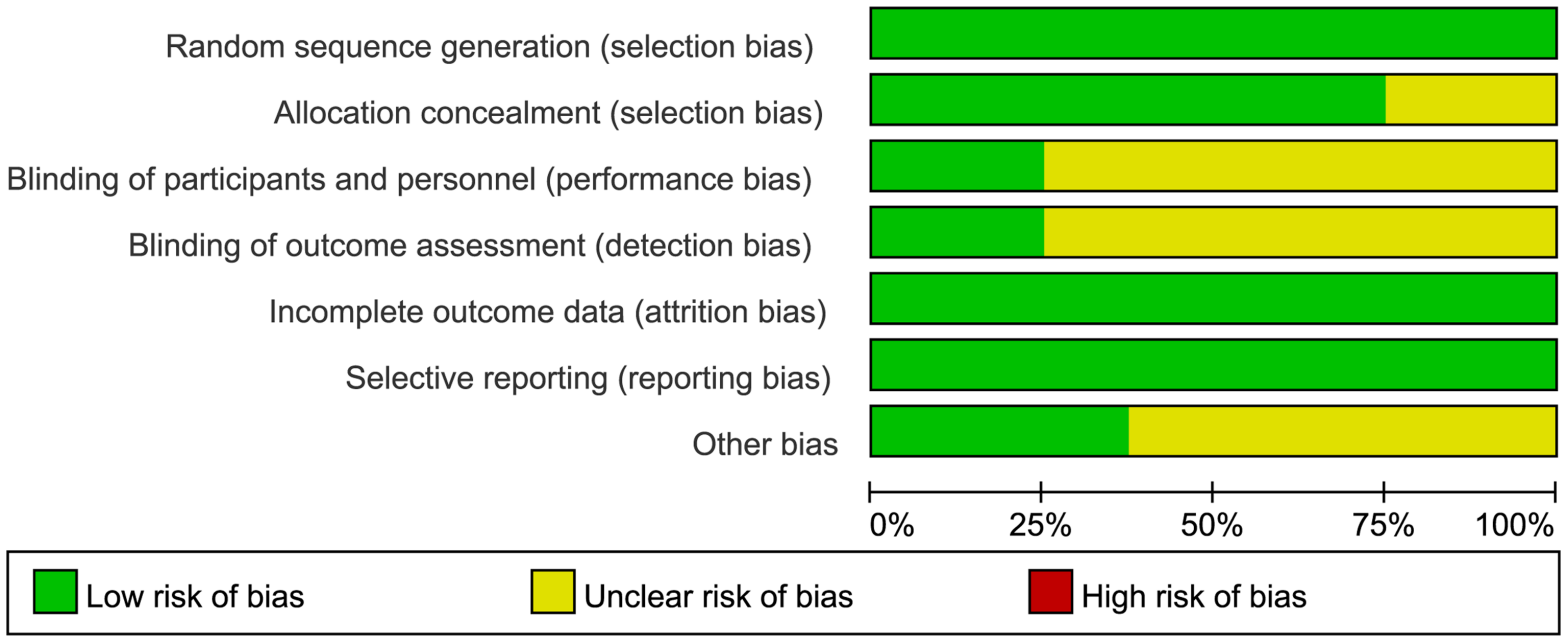
Risk of bias graph: review authors’ judgements about each risk of bias item, presented as percentages across all included studies.

**Figure 3:**
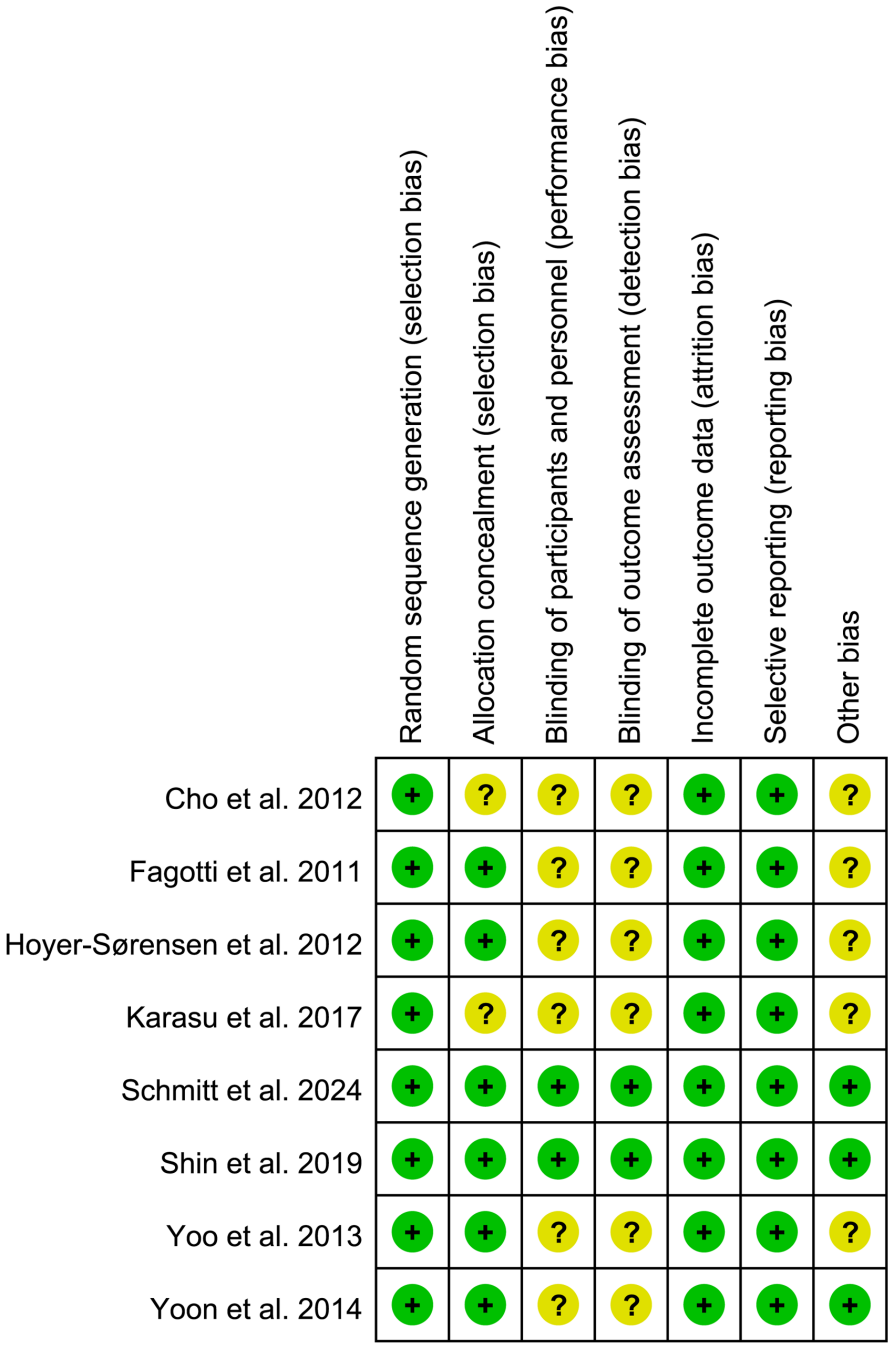
Risk of bias summary: review authors’ judgements about each risk of bias item for each included study.

For selection bias, all eight studies (20–27) reported adequate random sequence generation, and six studies (21, 22, 24–27) reported adequate allocation concealment. For blinding, two studies (24, 25) were assessed as having a low risk of performance bias, and the same two studies (24, 25) were considered at low risk of detection bias because they reported adequate blinding of outcome assessment. All included studies (20–27) showed a low risk of reporting bias, as all prespecified outcomes were reported. The detailed results of the bias risk assessment are illustrated in Figures 2, 3.

### Primary outcomes

**Figure 4:**
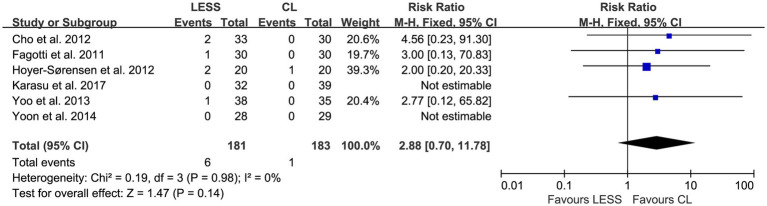
Forest plot of comparison: primary outcomes; outcome: perioperative complication rate.

A total of six studies (20–23, 26, 27), including 364 patients, reported the perioperative complication rate. No intraoperative complications were observed in either group. No statistically significant heterogeneity was detected among the studies (*p* = 0.98, *I*^2^ = 0%). A fixed effects model was utilized for pooling. The results revealed that there was no significant difference in the perioperative complication rate between the LESS and CL groups (RR, 2.88; 95% CI, 0.70 to 11.78; *p* = 0.14) (Figure 4).

**Figure 5:**
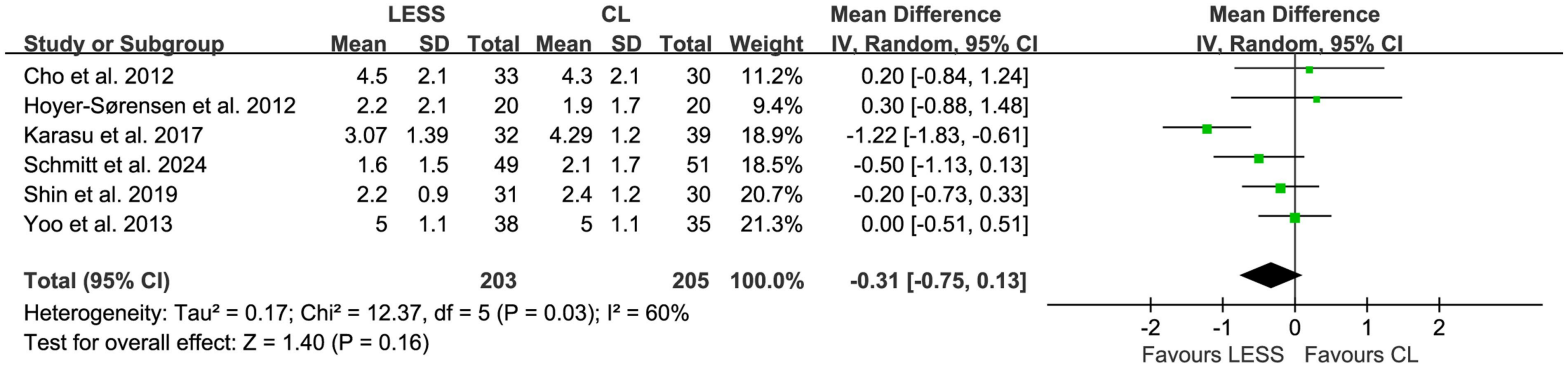
Forest plot of comparison: primary outcomes; outcome: postoperative pain scores at 6 h.

**Figure 6:**
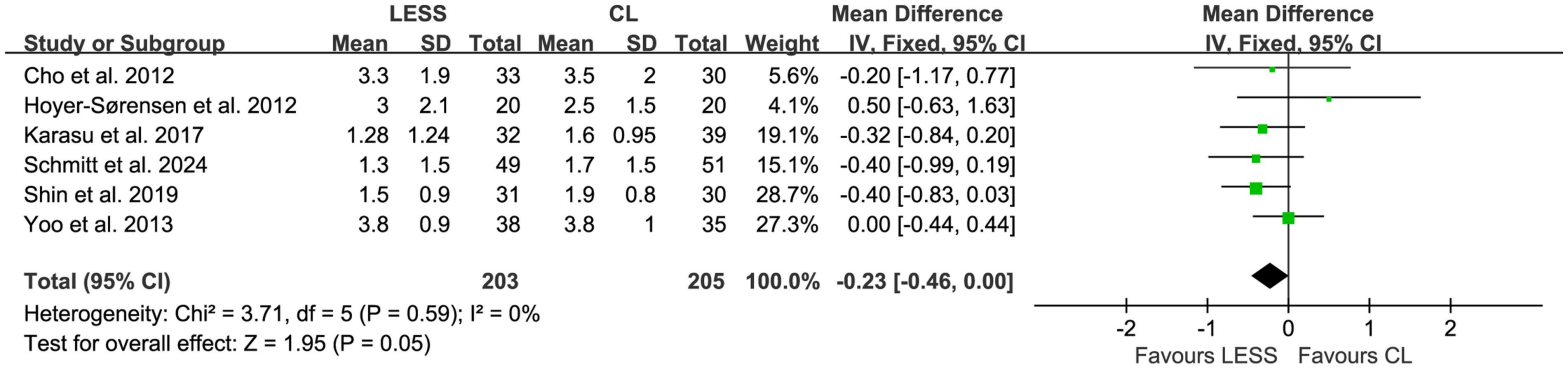
Forest plot of comparison: primary outcomes; outcome: postoperative pain scores at 24 h.

**Figure 7:**
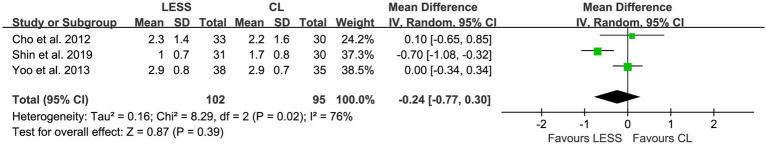
Forest plot of comparison: primary outcomes; outcome: postoperative pain scores at 48 h.

Meta-analyses were performed to evaluate postoperative pain scores at 6, 24, and 48 h. The results showed that there were no statistically significant differences in postoperative pain scores between the LESS and CL groups at 6 h (WMD, −0.31; 95% CI, −0.75 to 0.13; *p* = 0.16) (Figure 5), 24 h (WMD, −0.23; 95% CI, −0.46 to 0.00; *p* = 0.05) (Figure 6), and 48 h (WMD, −0.24; 95% CI, −0.77 to 0.30; *p* = 0.39) (Figure 7). Significant heterogeneity was observed among the studies for postoperative pain scores at 6 h (*p* = 0.03, *I*^2^ = 60%) and 48 h (*p* = 0.02, *I*^2^ = 76%), while no significant heterogeneity was found for postoperative pain scores at 24 h (*p* = 0.59, *I*^2^ = 0%).

In total, six studies (20–22, 24–26) reported cosmetic satisfaction, which was not analyzed in this meta-analysis because reports of this outcome were non-standardized in the included studies. Schmitt et al. (20) reported that self-reported scar satisfaction scale scores were similar between the two groups at 4 weeks after surgery. Yoo and Shim (22) found high satisfaction with cosmetic outcomes in both groups, with no significant difference in Manchester Scar Scale scores at 2 months after surgery. Pelosi and Pelosi (24) found that patients’ satisfaction with their scar at 1 month might be higher with LESS than with CL. Yoon et al. (25) showed that LESS was associated with significantly reduced scar formation and improved body image satisfaction at 4 months after surgery, compared to CL. Shin et al. (21) observed a statistically significantly higher rate of scar satisfaction in the LESS group than in the CL group, as reported by both patients and surgeons at discharge and 1 month after surgery. Jung et al. (26) reported that participants were asked about scar satisfaction using a numerical rating scale (NRS, ranging 0–10, with 10 representing maximum satisfaction) by a nurse who was blinded to treatment assignment. Scar satisfaction NRS scores showed no significant between-group differences at 1 month after surgery. However, scar satisfaction was significantly higher in the LESS group than in the CL group at 6 months and 1 year. Notably, variability in outcome measures and reporting methods prevented a formal meta-analytic synthesis of cosmetic satisfaction.

### Secondary outcomes

A total of five studies (20, 21, 23, 24, 27) reported the conversion rate. However, a meta-analysis could not be performed because no patients in either group required conversion to laparotomy. No placement of additional ports was reported in any patients across these studies.

**Figure 8:**
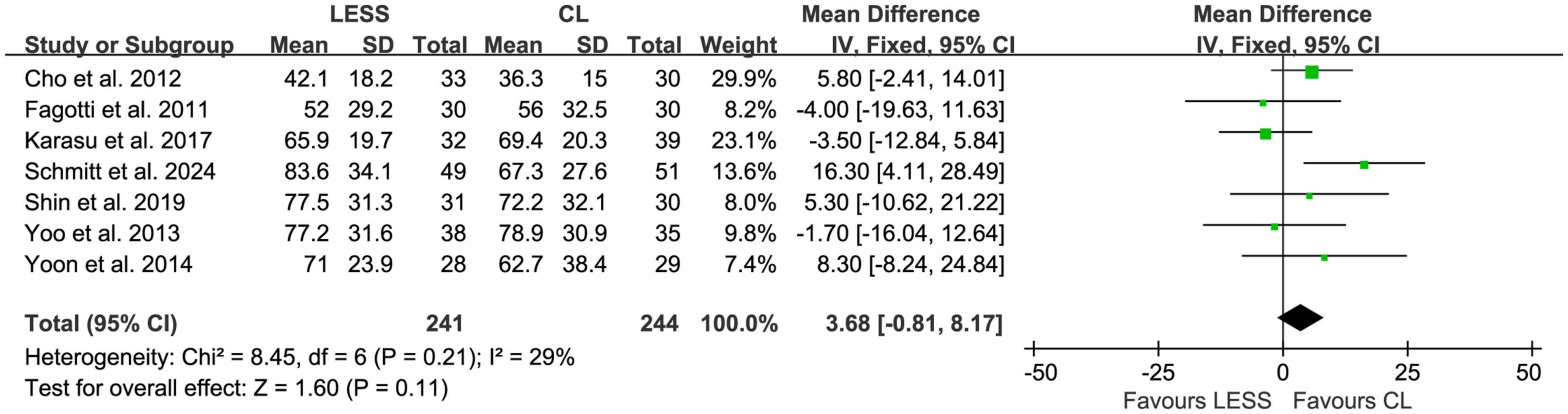
Forest plot of comparison: secondary outcomes; outcome: operative time.

Operative time was reported in seven studies (20, 21, 23–27), and no significant heterogeneity (*p* = 0.21, *I*^2^ = 29%) was detected among the studies. A fixed effects model was employed for pooling. The pooled data indicated no significant difference in operative time between the LESS and CL groups (WMD, 3.68; 95% CI, −0.81 to 8.17; *p* = 0.11) (Figure 8).

**Figure 9:**
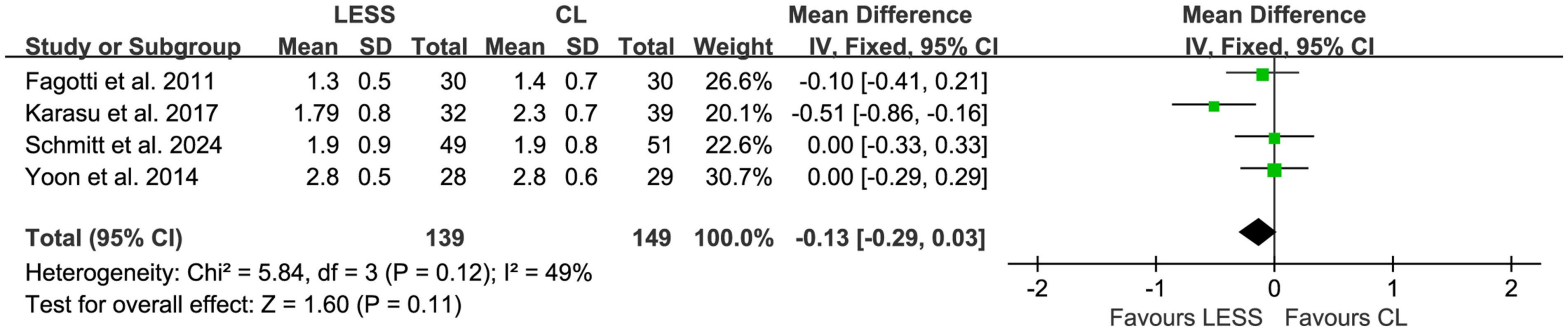
Forest plot of comparison: secondary outcomes; outcome: hospital stay.

Hospital stay after surgery was reported in four studies (21, 23, 24, 27), and no significant heterogeneity was detected among the studies (*p* = 0.12, *I*^2^ = 49%). A fixed effects model was utilized for pooling. The pooled data indicated no significant difference in hospital stay after surgery between the LESS and CL groups (WMD, −0.13; 95% CI, −0.29 to 0.03; *p* = 0.11) (Figure 9).

**Figure 10:**
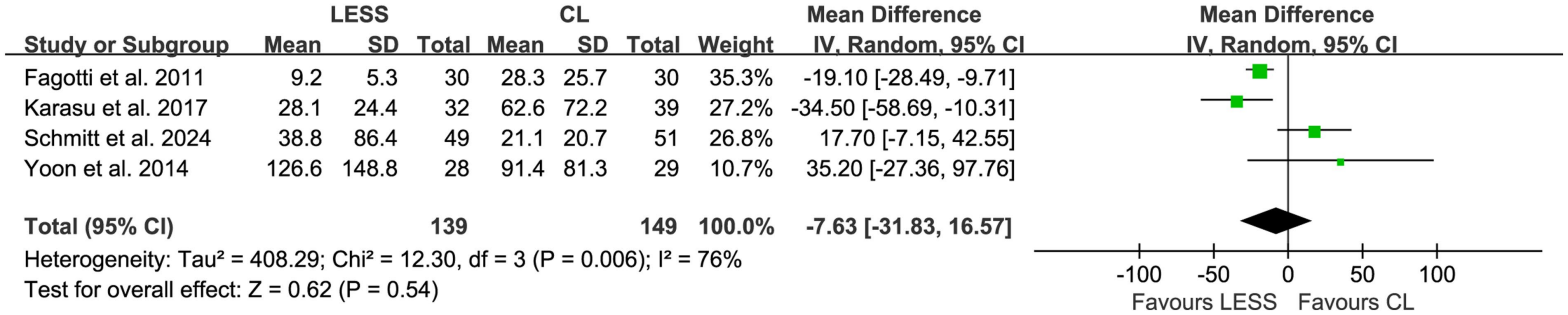
Forest plot of comparison: secondary outcomes; outcome: estimated blood loss during surgery.

A total of four studies (21, 23, 24, 27), including 288 patients, provided data on estimated blood loss during surgery. There was statistically significant heterogeneity among the studies (*p* = 0.006, *I*^2^ = 76%). The pooled data, analyzed using a random effects model, showed that there was no significant difference in estimated blood loss during surgery between the LESS and CL groups (WMD, −7.63; 95% CI, −31.83 to 16.57; *p* = 0.54) (Figure 10).

**Figure 11:**
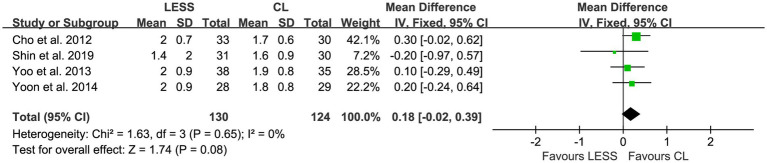
Forest plot of comparison: secondary outcomes; outcome: hemoglobin drop.

Hemoglobin drop was reported in four studies (20, 25–27), and no significant heterogeneity (*p* = 0.65, *I*^2^ = 0%) was detected among the studies. A fixed effects model was utilized for pooling. The pooled data indicated no significant difference in hemoglobin drop between the LESS and CL groups (WMD, 0.18; 95% CI, −0.02 to 0.39; *p* = 0.08) (Figure 11).

### Subgroup analysis

We performed subgroup analyses for estimated blood loss during surgery and postoperative pain scores at 6 h using a limited number of studies, based on the type of sample size and study location. There were no significant differences in the subgroup analyses compared to the original results for either estimated blood loss during surgery or postoperative pain scores at 6 h (Supplementary Figures 1–4). Subgroup analysis was not performed for postoperative pain scores at 48 h because of insufficient data.

### Sensitivity analysis

A sensitivity analysis was conducted for estimated blood loss during surgery and postoperative pain scores at 6 and 48 h. The analysis demonstrated that the result for estimated blood loss during surgery was robust because none of the individual studies substantially influenced the pooled effect. Although sensitivity analyses were conducted by repeating the whole analysis after excluding one study (23) with an unclear risk of bias for postoperative pain scores at 6 h and one study (25) with an unclear risk of bias for postoperative pain scores at 48 h, the results remained unchanged.

## Discussion

The potential of LESS in treating benign adnexal diseases lies in its minimally invasive nature and swift recovery. Ever since its introduction in gynecology by Paek et al. (28), LESS has been reported for ectopic pregnancy, adnexal surgery, hysterectomy, and gynecologic malignancies (6–9, 29–31). However, LESS can be challenging, even frustrating, to perform because of reduced visualization, loss of triangulation, and instrument interference (32). Recent technological advancements have provided the opportunity to revisit the concept of laparoscopic surgery limited to the use of a single incision (33). In the last decade or so, several studies have been published on the safety and efficacy of LESS for benign adnexal diseases. However, it remains unclear whether LESS provides additional advantages compared to CL for benign adnexal diseases. Generally, meta-analyses of RCTs can summarize the results of independent studies to provide the highest level of evidence. In this meta-analysis, we included eight RCTs with a total of 525 patients to compare the safety and efficacy of LESS with CL for benign adnexal diseases.

In this systematic review and meta-analysis, our findings confirmed that LESS was generally equivalent to CL for benign adnexal diseases in terms of intraoperative complications, postoperative complications, operative time, estimated blood loss during surgery, hemoglobin reduction after surgery, conversion to laparotomy, and hospital stay after surgery. These results are consistent with a previous meta-analysis that compared LESS with CL for gynecologic procedures, including hysterectomy and adnexal surgery (34). A meta-analysis of LESS appendectomy, which included nine studies, also showed that LESS has perioperative outcomes comparable to CL (35).

Safety is one of the most important considerations when a new surgical technique is developed. The safety of LESS was compared with that of CL by analyzing perioperative complication rates. In the included studies, no intraoperative complications were reported in either the LESS group or the CL group. The incidence of perioperative complications was low in both groups, and the pooled data showed no significant difference (RR, 2.88; 95% CI, 0.70 to 11.78; *p* = 0.14). The wide confidence interval reflects imprecision, likely due to limited statistical power from small sample sizes, heterogeneous reporting of complications, and low event rates. These limitations warrant cautious interpretation and underscore the need for larger studies with standardized outcome definitions. No study provided long-term data on port-site herniation rates. One study (26) provided 1-year follow-up data on incisional hernia, which showed no cases in either group. LESS is not easy to perform: The surgeon (even if experienced in CL) must overcome obstacles such as difficulties in establishing the field of vision during surgery, instrument crowding, loss of triangulation, and the challenging suturing procedure. Despite these technical challenges, this systematic review and meta-analysis demonstrated that LESS for benign adnexal diseases is comparable to CL in both efficacy and safety.

Postoperative pain is a critical issue because of its connection to postoperative analgesic use, hospital stay, and return to normal daily activities. In this meta-analysis, postoperative pain at 6, 24, and 48 h showed no statistically significant difference between the LESS and CL groups, in agreement with findings from other meta-analyses in the literature (30−33). A meta-analysis of LESS cholecystectomy, including 858 participants from 11 RCTs, showed no difference in postoperative pain at 6 and 24 h between the LESS and CL groups (33). It is difficult to compare postoperative pain related to certain procedures, and several factors can influence the results, such as the amount of residual gas in the abdomen, the temperature of the gas and the irrigation fluid used, coagulative or direct tissue trauma, the number and size of ports used, and patient-related factors (26).

A potential cosmetic benefit of LESS compared to CL has been suggested, mainly due to the single umbilical incision that results in a more concealed scar (34). A meta-analysis of RCTs comparing single-incision versus conventional laparoscopic appendectomy showed that the single-incision group was associated with better cosmetic results (35). In the present meta-analysis, six studies reported cosmetic satisfaction, but quantitative synthesis was not performed owing to heterogeneous assessment tools and non-standardized reporting across the studies. Pelosi and Pelosi et al. (24), Shin et al. (21), and Yoon et al. (25) revealed that the LESS group reported significantly higher cosmetic satisfaction than the CL group, whereas Schmitt et al. (20) and Yoo et al. (22) found no difference in the cosmetic outcome between the two groups. Jung et al. (26) reported that scar satisfaction at 1 month showed no significant difference between the two groups, whereas scar satisfaction was significantly higher in the LESS group than in the CL group at 6 months and 1 year. As previously highlighted (35), cosmetic satisfaction should be assessed in the long term as it could depend on the time of the survey.

### Limitations

The present meta-analysis has several limitations. First, only eight RCTs with small sample sizes were included, which may compromise the reliability of the findings. Although our analysis represents the most comprehensive synthesis of available RCT data to date, non-significant results (e.g., complication and conversion rates) should be interpreted cautiously. Second, the limited number of included studies made it impractical to evaluate potential publication bias. Third, the included studies did not provide appropriate data to compare cosmetic satisfaction between LESS and CL. Fourth, significant heterogeneity was observed in pain scores at both 6 and 48 h, as well as in estimated blood loss. This substantial heterogeneity likely reflects clinical variability, including institutional variations in surgical practices, patient-related risk factors, and differences in methods used for estimating blood loss (e.g., gravimetric or suction measurement) and assessing pain (using VAS or NRS scales). Fifth, owing to limited data availability, we were unable to assess how heterogeneity in the management of benign adnexal diseases—including cystectomy, adnexectomy, and endometrioma excision—impacts the primary and secondary outcomes. Failure to account for such variability may have introduced confounding bias and thereby restricted the interpretability of our findings. Sixth, some included studies were assessed as having an unclear risk of bias, particularly in terms of blinding, which may potentially affect study validity. These biases warrant cautious interpretation of the results; blinding-related bias may have overestimated intervention effects and reduced estimation accuracy. Future studies should optimize blinding methods and perform sensitivity analyses. Finally, the follow-up periods in the included studies were generally short, so long-term clinical outcomes—such as port-site hernia, cosmetic satisfaction, and chronic postoperative pain—remain to be fully elucidated. Future prospective studies with standardized long-term follow-up protocols (e.g., ≥5 years) are urgently needed to evaluate these outcomes.

## Conclusion and implications

In conclusion, this systematic review and meta-analysis demonstrated that LESS is generally equivalent to CL with respect to perioperative complication rates, postoperative pain scores at 6, 24, and 48 h, operative time, hospital stay after surgery, and estimated blood loss during surgery. In selected female patients with benign adnexal diseases, LESS appears to be a safe and feasible alternative to CL. However, additional large-scale, long-term prospective randomized trials are required to confirm whether LESS truly offers benefits, including better cosmetic satisfaction, compared to CL for benign adnexal diseases.

## Data Availability

The original contributions presented in the study are included in the article/Supplementary material, further inquiries can be directed to the corresponding authors.

## Supplementary material

SUPPLEMENTARY FIGURE 1 Forest plots of estimated blood loss during surgery in subgroup analysis based on sample size.

SUPPLEMENTARY FIGURE 2 Forest plots of estimated blood loss during surgery in subgroup analysis based on study location.

SUPPLEMENTARY FIGURE 3 Forest plots of postoperative pain scores at 6 h in subgroup analysis based on sample size.

SUPPLEMENTARY FIGURE 4 Forest plots of estimated postoperative pain scores at 6 h in subgroup analysis based on study location.
