# Supplementary figures and images for: Laparoendoscopic single-site surgery compared with conventional laparoscopy for benign adnexal diseases: a systematic review and meta-analysis of randomized controlled trials
Source: Front Med (Lausanne). 2026 Apr 9;13:1779247. doi: 10.3389/fmed.2026.1779247 (PMC13102826; doi:10.3389/fmed.2026.1779247)

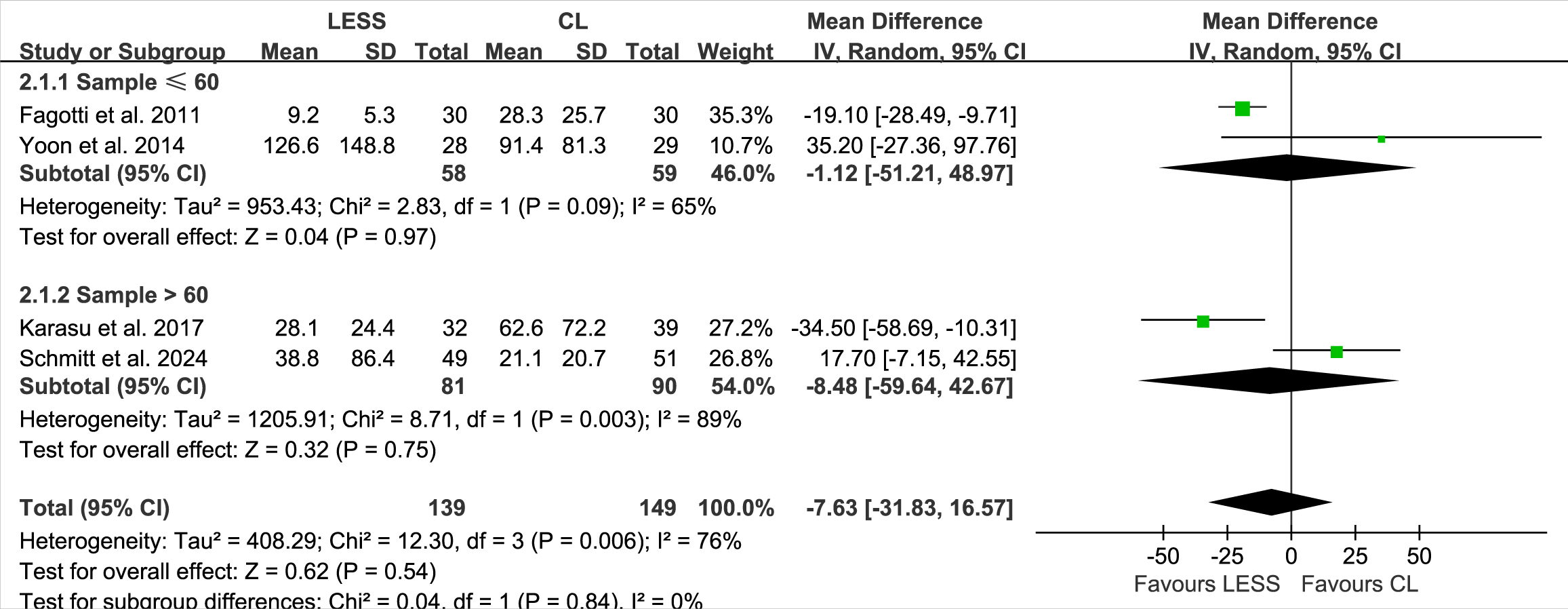

Supplement: SUPPLEMENTARY FIGURE 1 — Forest plots of estimated blood loss during surgery in subgroup analysis based on sample size. [file Image_1.tif]

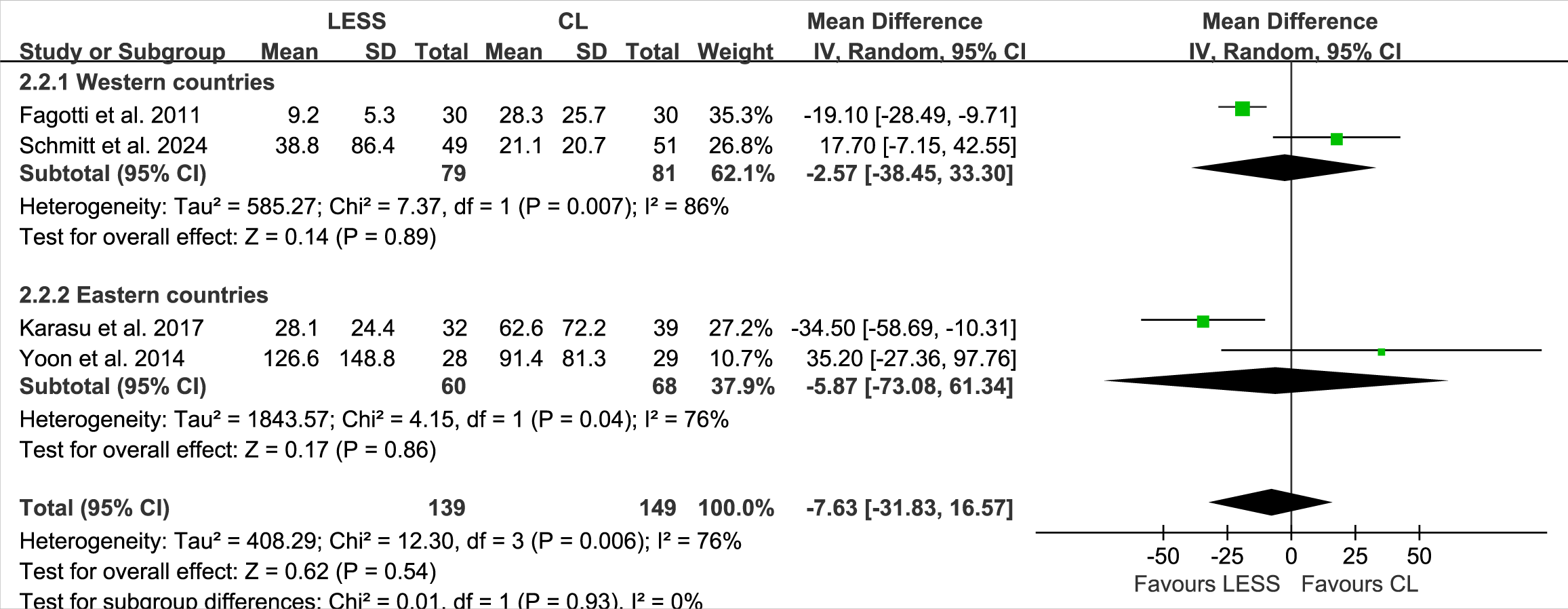

Supplement: SUPPLEMENTARY FIGURE 2 — Forest plots of estimated blood loss during surgery in subgroup analysis based on study location. [file Image_2.tif]

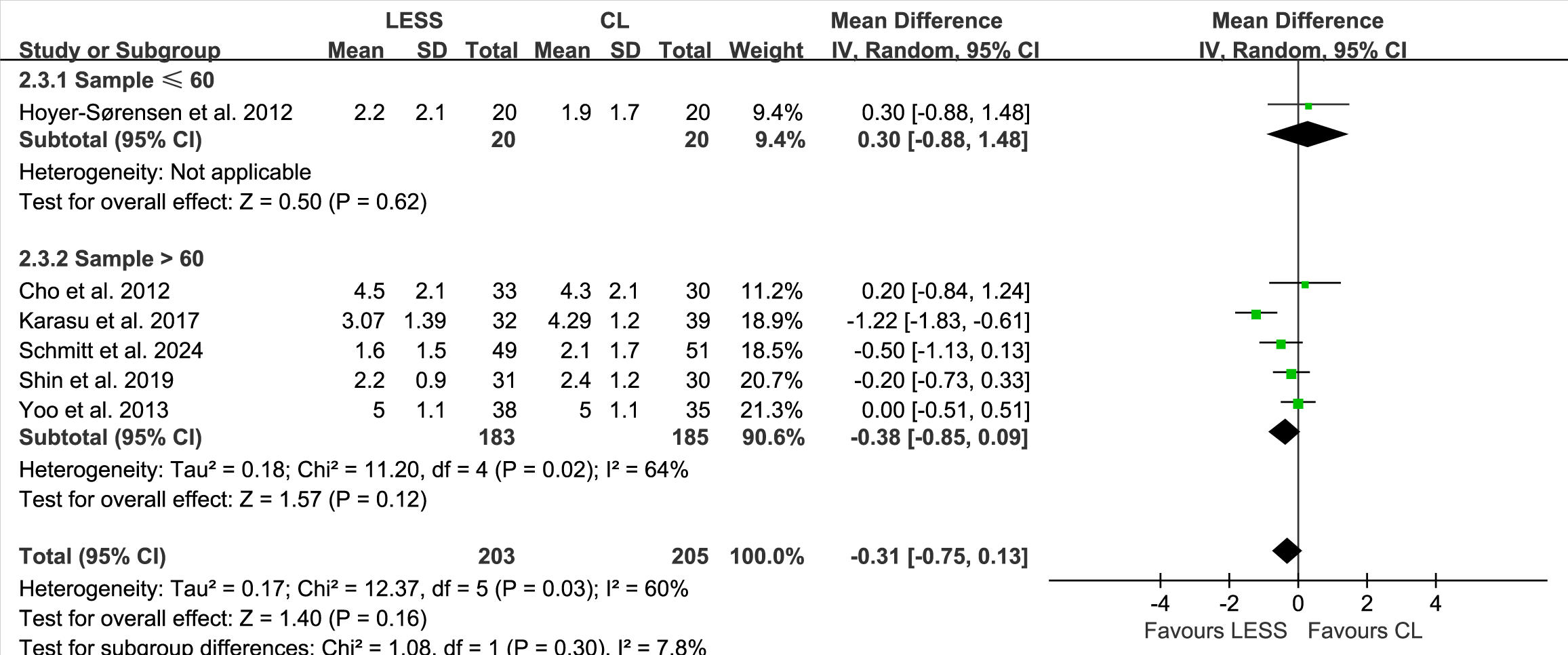

Supplement: SUPPLEMENTARY FIGURE 3 — Forest plots of postoperative pain scores at 6 h in subgroup analysis based on sample size. [file Image_3.tif]

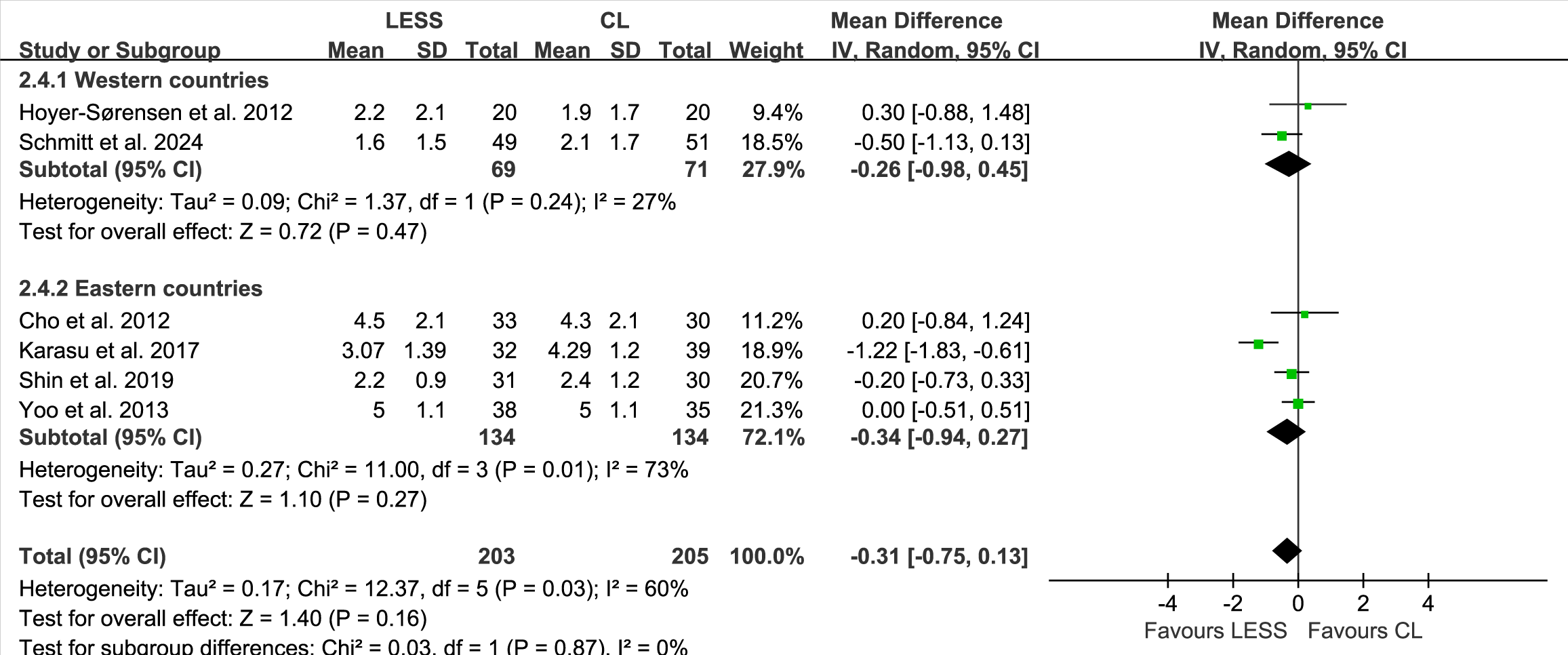

Supplement: SUPPLEMENTARY FIGURE 4 — Forest plots of estimated postoperative pain scores at 6 h in subgroup analysis based on study location. [file Image_4.tif]
